# The Climatic Resilience of the Sasanian Empire

**DOI:** 10.1007/s10745-024-00554-w

**Published:** 2025-01-13

**Authors:** Matthew J. Jacobson, Alison L. Gascoigne, Dominik Fleitmann

**Affiliations:** 1 Division of Agrarian History, Department of Urban and Rural Development, Swedish University of Agricultural Sciences (SLU), Ulls Väg 27, 756 51 Uppsala, Sweden; 2 Archaeology, Faculty of Arts and Humanities, Avenue Campus, University of Southampton, SO17 1BF, Southampton, United Kingdom; 3 Department of Environmental Sciences, University of Basel, Bernoullistrasse 30-32, 4051 Basel, Switzerland

**Keywords:** Paleoclimate, Late antiquity, Resilience, Climate-society interactions, Water infrastructure, Archaeology, Sasanian empire

## Abstract

The Sasanian Empire (224–651 CE) has been given relatively little attention in research on climate-society interactions when compared to the neighboring Byzantine Empire, despite evidence of changing conditions and an agricultural economy that is theoretically vulnerable to droughts due to low annual precipitation. We review the available historical, archaeological, paleo-environmental, and paleo-climatic evidence to assess whether climatic conditions factored into periods of Sasanian growth and decline. We find evidence for drier conditions across Sasanian territories at the turn of the sixth century, a pattern that extends to the Aegean, Anatolia, and Central Asia. These same conditions contributed to a significant decline for the nearby Kingdom of Himyar but occurred alongside a period of expansion and intensification for the Sasanian Empire. We suggest that a combination of careful management of water infrastructure, including qanats, which can conserve water resources during dry periods, and land-use strategies that are both diverse and flexible, may have mitigated the worst impacts of this dry period. However, we note several weaknesses in the available data that still hinder confident interpretations of the potential impacts of climate change in the Sasanian Empire. Notably, there are gaps in the coverage of paleo-hydrological records and a complete lack of terrestrial paleo-temperature records in the region, as well as low resolution and high chronological uncertainties in the archaeological and paleo-environmental evidence.

**Supplementary Information:**

The online version contains supplementary material available at 10.1007/s10745-024-00554-w.

## Introduction

**Fig. 1:**
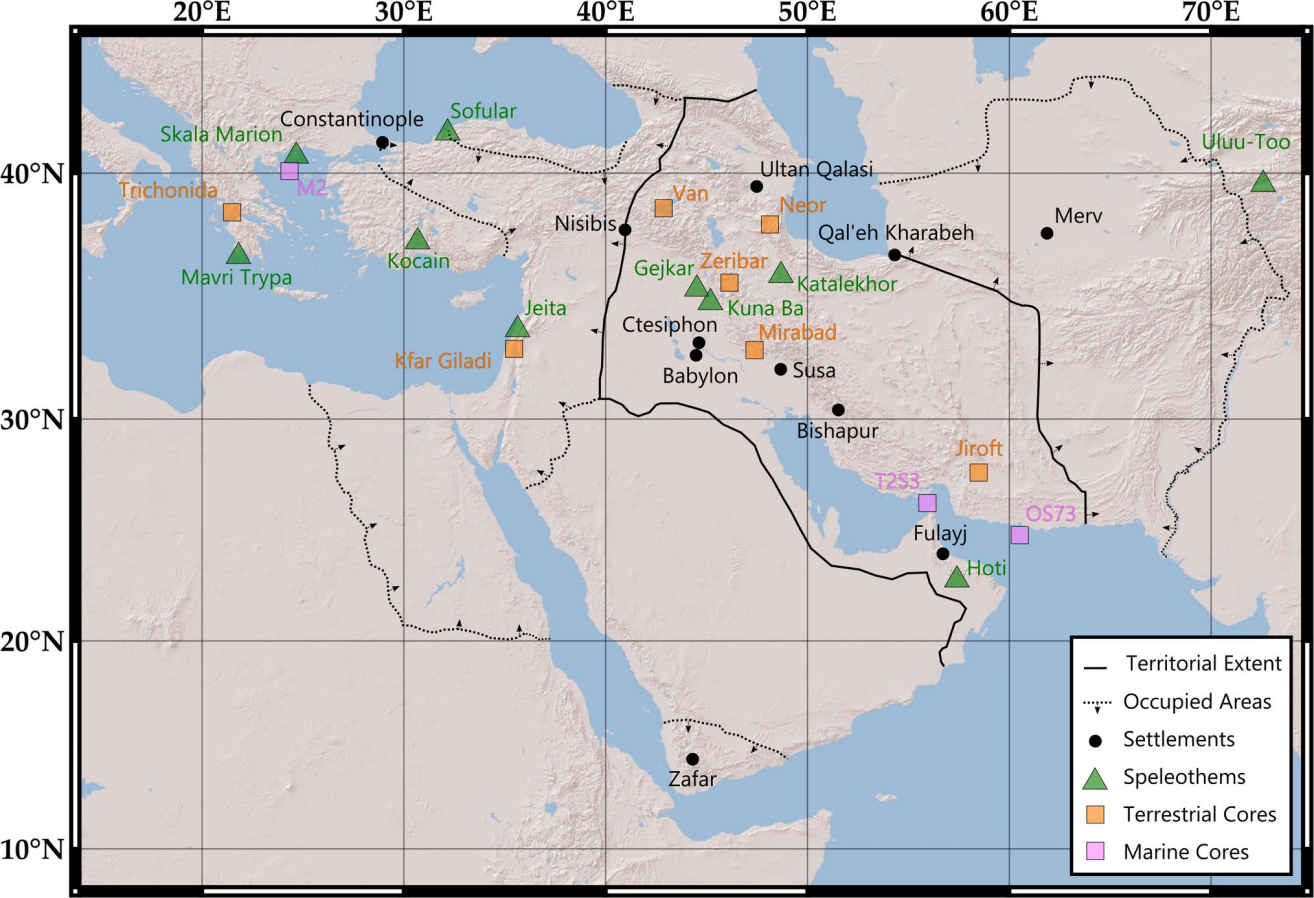
Map showing the Sasanian Empire, with paleoclimate archives and locations mentioned in the text

**Fig. 2:**
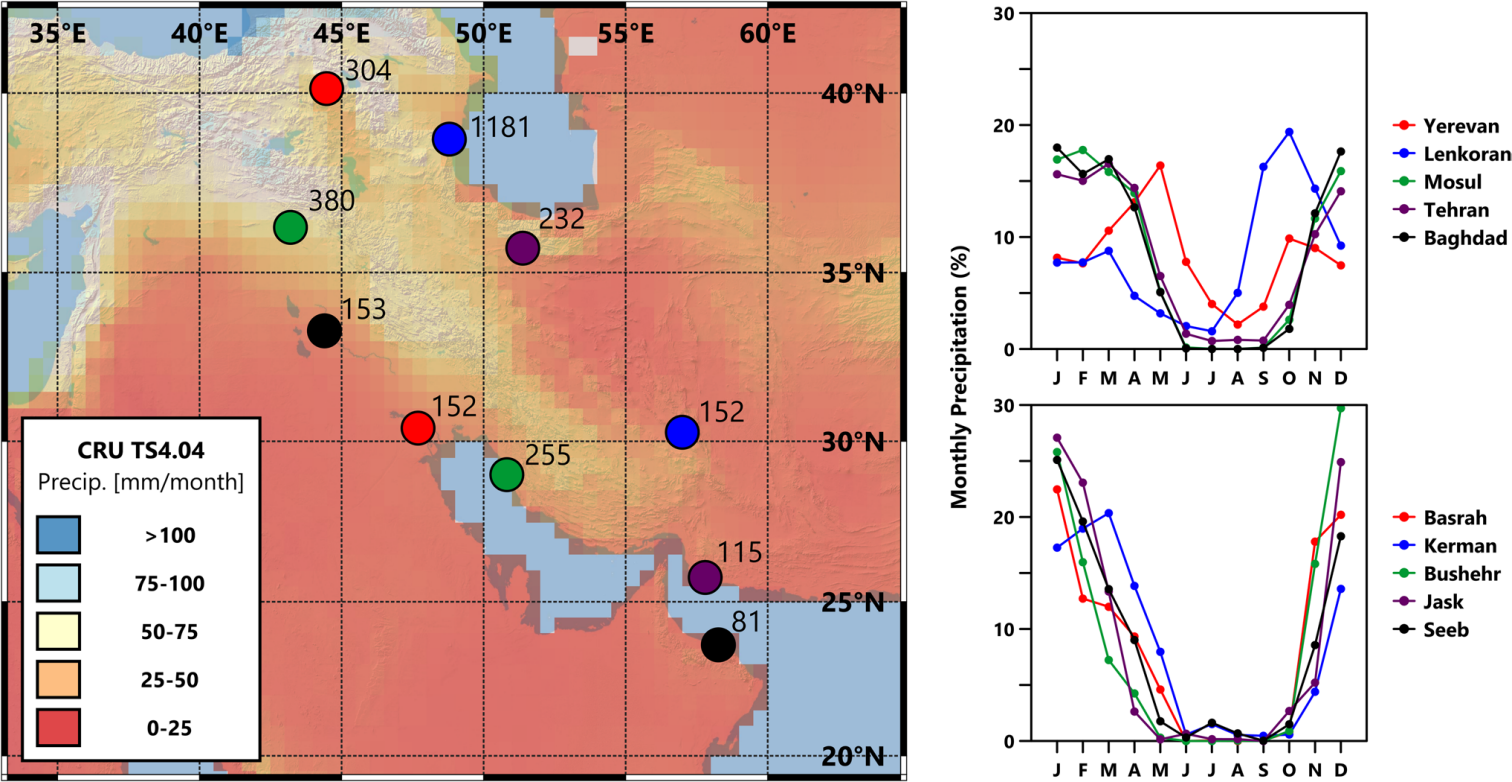
Modern precipitation data. The map shows CRU TS4.04 (1901–2021) averaged monthly precipitation data (University of East Anglia Climatic Research Unit et al., 2022). Colored circles represent weather stations, labelled with their average annual precipitation (in mm) and matching with their seasonality of precipitation graphs (as percentages, right) (data from Peterson & Vose, 1997).

In the past decade, scholarship on the societal impacts of climate change in Late Antiquity (third-seventh centuries CE) has been growing, particularly in SW Asia (e.g., McCormick et al., 2012; Haldon et al., 2014; Fleitmann et al., 2022). Large-scale climate changes, such as the Dark Ages Cold Period (DACP: c. 450–800 CE) and Late Antique Little Ice Age (LALIA: c. 536–660 CE), have been argued to influence agricultural productivity, with consequent impacts on the economy, migrations, conflict, and the end of empires (e.g., Büntgen et al., 2016 and above references). Matloubkari and Islam (2022) have reasoned that the Sasanian Empire, which dominated Persia (Fig. 1) in a period roughly coincident with Late Antiquity (224–651 CE), was severely influenced by climatic conditions. Based on prior research, however, we reached a different conclusion. We argue that this topic requires a full assessment for several reasons. First, despite a total of nine paleoclimate records detailing conditions for the Sasanian Empire, previous research has been limited to temporal correlations and/or environmentally deterministic comments about “collapse” (e.g., Sharifi et al., 2015; Peregrine, 2020; Hoyer et al., 2023). Simplistic comparisons have long been comprehensively critiqued, with the establishment of causal links and examinations of societal resilience now considered crucial for successful studies of human-climate interactions (Coombes & Barber, 2005; Haldon & Rosen, 2018; Moreland, 2018; Degroot et al., 2021). We use the definition of climate resilience from ecology adopted by the IPCC that it is the ability of a system to retain or rapidly restore its essential functions and persist during periods of climate change (IPCC, 2012). Second, significant climate shifts have been identified in paleoclimate proxies in SW Asia and surrounding regions, which contributed to societal change in the contemporaneous Byzantine Empire (Haldon et al., 2014; Izdebski et al., 2016; Jacobson et al., 2022) and the Kingdom of Himyar (Fleitmann et al., 2022). Likely, the Sasanian Empire would also have experienced these events. Third, climate change in this region has been argued to have caused societal change in other historical periods (Altaweel et al., 2019; Sinha et al., 2019) and recent geo-political instability (Gleick, 2014; Kelley et al., 2015; Flohr et al., 2017). Finally, agriculture was the key component of the economy (Seyf, 2006) and the empire contains some of the driest and hottest places on earth (Mildrexler et al., 2006; Djamali et al., 2011), including regions with annual precipitation close to the minimum threshold for rain-fed agriculture (250 mm/yr^−1^ according to Hole, 2007) (Fig. 2). Even small hydro-climatic fluctuations thus had the potential to cause significant impacts.

We present an interdisciplinary investigation into climatic, agricultural, and societal change during the Sasanian Empire and an assessment of the quality of available historical, archaeological, paleoenvironmental, and paleoclimatic evidence. Specifically, we present a history of climate changes and consider their relevance for periods of Sasanian growth and decline.

## Materials and Methods

### Paleoclimate Records

Paleoclimate proxy records provide indirect evidence for climatic conditions in the past, usually relating to precipitation and/or temperature. Nine available continental paleo-hydrological archives in Sasanian territory provide evidence of conditions during the Sasanian period (Table 1). Here, we focus on hydrological conditions due to the regional importance of effective moisture in determining potential agricultural productivity and the paucity of paleo-temperature archives. The hydrological and erosive conditions in arid environments lead to scarce and unevenly distributed archives, mainly due to their requirement for adequate effective moisture to form (Burstyn et al., 2019). Eight of the nine available records are thus located in the western regions around the Fertile Crescent (Fig. 1), and all records are found at high elevations (> 650 m asl). These comprise four speleothem records and five sediment sequences: four from lakes and one from a peat deposit. Both speleothems – cave deposits such as stalagmites – and sediments record past climatic conditions during deposition in their geochemistry and physical properties (Jones et al., 2019). These records are grouped according to the frequency of their data points, with four deemed high-resolution (Gejkar, Hoti, Kuna Ba, Neor: 2.2–3 years between samples) while the remainder are low-resolution (Jiroft, Katalekhor, Mirabad, Van, Zeribar: 67–333 years between samples). We utilize the high-resolution records to assess short-term fluctuations in climatic conditions and all records to assess long-term trends and differences between centuries. It is important to note that all records have age uncertainties of ± multiple decades, which are especially challenging when assessing short-term fluctuations. While all utilized proxy records are paleo-hydrological (i.e., a large portion of their variability is determined by regional/local rainfall), each has a slightly different interpretation due to effects during formation (Table 1). Most records here are produced from δ^18^O: ratios between heavier ^18^O and the lighter ^16^O oxygen isotopes. A significant influence on this ratio in sediment sequences and speleothems is the “amount effect”, a negative correlation between the amount and δ^18^O of precipitation (Dansgaard, 1964). The ratios are then further modulated by the amount of water entering the lake or cave system, meaning δ^18^O ratios can more specifically be interpreted as lake water balance (input vs. output) and cave drip rates. This means that for some lakes, evaporation is a more significant influence on δ^18^O ratios than the “amount effect.” It is important to note that all δ^18^O records in this region have a winter-spring seasonal bias due to precipitation seasonality. The two other records use Titanium (Ti) measurements as a proxy for dust influx and aridity. Greater amounts of Ti signify an increase in Aeolian (wind-blown) dust input to the region, which will occur during drier conditions (Sharifi et al., 2015; Vaezi et al., 2022).

**Table 1:** Paleo-hydrological records in Sasanian Persia

Archive	Type	Proxy	Interpretation^1^	Resolution^2^	Ref(s)
Gejkar	Cave	δ^18^O	Effective Moisture	**2.17**	(Flohr et al., 2017)
Hoti	Cave	δ^18^O	Precipitation Amount	**2.56**	(Fleitmann et al., 2022)
Jiroft	Peat	Ti/Al	Dust Influx/Aridity	66.67	(Vaezi et al., 2022)
Katalekhor	Cave	δ^18^O	Precipitation Amount	66.67	(Andrews et al., 2020)
Kuna Ba	Cave	δ^18^O	Precipitation Amount	**2.98**	(Sinha et al., 2019)
Mirabad	Lake	δ^18^O	Precipitation Amount/Seasonality	333.33	(Stevens et al., 2006)
Neor	Lake	Ti	Dust Influx/Aridity	**2.99**	(Sharifi et al., 2015)
Van	Lake	δ^18^O	Humidity	125	(Wick et al., 2003; Barlas Şimşek & Çağatay, 2018)
Zeribar	Lake	δ^18^O	Precipitation Amount/Seasonality	100	(Stevens et al., 2001)

^1^Interpretation comes from the original publication of each record

^2^Years between samples averaged for the first millennium CE. High-resolution archives in bold

From previously published paleo-hydrological proxy data, we calculated z-scores, which are standardized units that enable simpler comparison of variables on different scales, as follows:

$$\:\text{Z}=\frac{\text{v}\text{a}\text{l}\text{u}\text{e}-\text{m}\text{e}\text{a}\text{n}}{standard\:deviation}$$

We calculated centurial averages for each record and decadal averages for the four high-resolution archives to simplify statistical comparison. In the records we discuss here, positive (negative) z-scores represent conditions drier (wetter) than mean conditions. We also calculated a mean z-score for each resolution, which broadly characterizes climatic conditions across Sasanian Persia. However, as climatic conditions can be heterogeneous on a regional level (e.g., Jacobson et al., 2021), this calculated variable may obfuscate paleo-hydrological change on smaller spatial scales and should be used cautiously.

## Evidence for Agricultural and Human Impacts

**Fig. 3:**
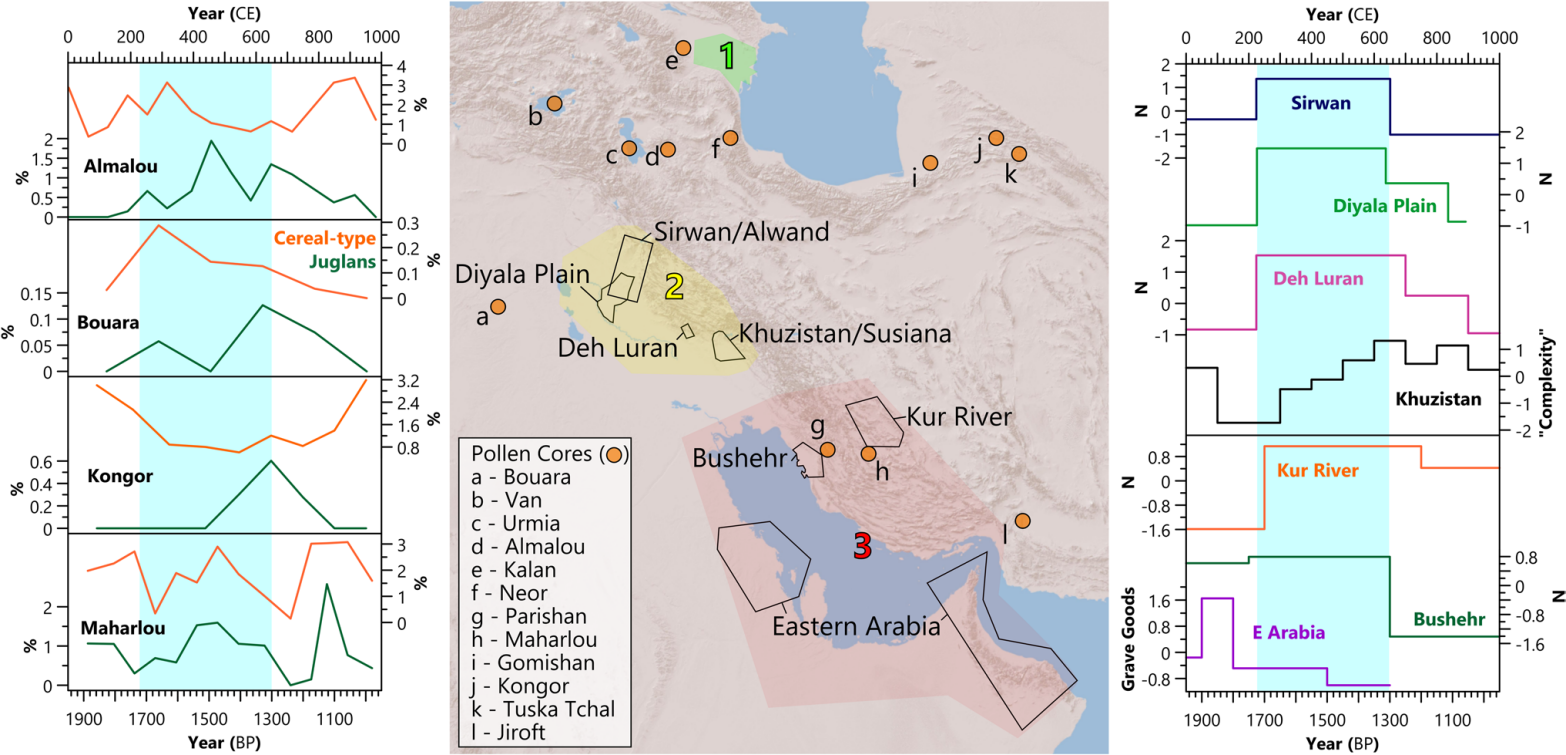
Locations of and data from pollen cores (left) and archaeological surveys (right). It is important to consider that the pollen data has significant dating uncertainties that are not visualized here. Key regions highlighted (1) the Mughan Steppe, (2) Lower Mesopotamia, and (3) the Persian Gulf. Archaeological survey results are presented as z-scores. Blue highlighted sections represent the Sasanian period: 224–651 CE. References for datasets can be found above

To investigate past vegetation and agriculture, we use palynological (pollen) data and information extracted from lake or peat sediments in the Sasanian Empire (Fig. 3). To synthesize the data from different sites, we extracted the percentages of cereal-type and walnut pollen (representative of the intensity of agriculture and arboriculture, respectively) from the four records with > two samples during the Sasanian Period and examined the changes over time during the first Millennium. These records are from Lakes Almalou (Djamali, de Beaulieu, Andrieu-Ponel et al., 2009), Bouara (Gremmen & Bottema, 1991), Kongor (Shumilovskikh et al., 2016), and Maharlou (Djamali, de Beaulieu, Miller et al., 2009; Saeidi Ghavi Andam et al., 2020). We discuss records with fewer samples or without publicly available data but do not plot them. These are from Gomishan (Leroy et al., 2013), Jiroft (Vaezi et al., 2022), Kalan (Ramezani et al., 2021), Neor (Ponel et al., 2013), Parishan (Jones et al., 2015; Djamali et al., 2016), Tuska Tchal (unpublished, but discussed in Shumilovskikh et al., 2017), Urmia (Talebi et al., 2016), and Van (Van Zeist & Woldring, 1978).

To examine past societal changes, we collated settlement counts and other data from previous archaeological projects in the territory of the Sasanian Empire (Fig. 3): Sirwan/Alwand (Casana & Glatz, 2017), the Diyala Plain (Adams, 1965), Deh Luran (Neely, 2016), Kur River basin (Hartnell, 2014), Bushehr (Carter et al., 2006) and Eastern Arabia (Kennet, 2007). We also extracted data from the Seshat Global History Databank (https://seshatdatabank.info/), a systematic synthesis of nine multi-variate characteristics for numerous polities based on archaeological and historical evidence. The Khuzistan/Susiana region is one of the databank’s case studies, with nine variables assessed for each century: polity population, polity territory, capital population (i.e., Ctesiphon for the Sasanian Empire), hierarchical levels, government complexity, infrastructure, information systems (i.e., record keeping), texts (i.e., publishing of literature), and sophistication of currency (Turchin et al., 2015). Turchin et al. (2017) previously conducted Principal Component Analysis (PCA) on this dataset and extracted a variable (PC1) that reflects societal “complexity.” Though historical quantification in such projects suffers from inherent subjectivity and value judgments and will require updating as future research is conducted, the record is helpful in contextualizing and summarizing current understanding of broad societal change (Turchin et al., 2012; Turchin, 2018; Slingerland et al., 2020).

## Results and Discussion

###  Climate Change

**Fig. 4:**
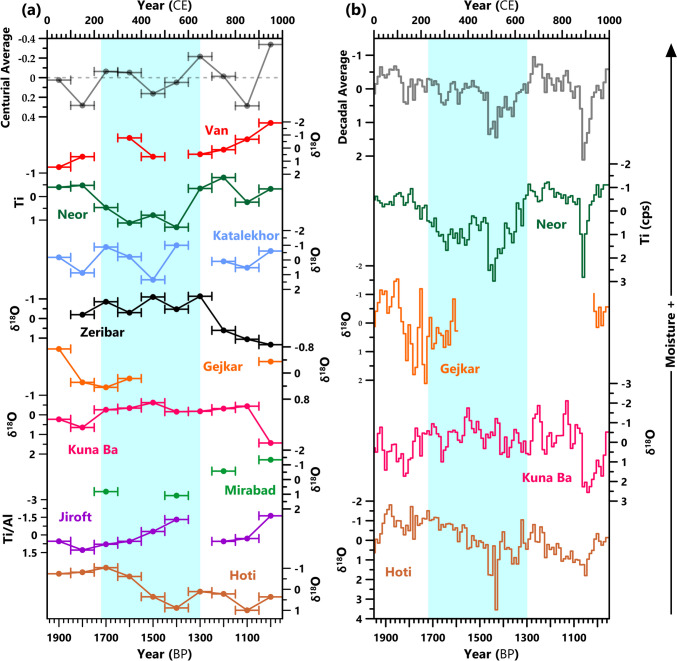
Paleo-hydrological data from the Sasanian Empire. (a) Centurial and (b) decadal z-scores for each record, aligned from N-S. Average z-scores of all records are displayed at the top of both graphs; the decadal average was calculated using only the high-resolution records. Considering that these datasets have varied age uncertainties that are not visualized here is important. Full datasets are visualized in Supplementary Fig. S1. References for datasets can be found in Table 1.

The paleo-hydrological proxy records reveal significant spatial and temporal variability during the first Millennium CE (Fig. 4). Prior to the Sasanian Period, evidence suggests a wetter first century and drier second century in all regions but the north. This pattern is most exaggerated in the Gejkar δ^18^O record, which suggests wet conditions peaking at 30–110 CE and dry conditions at 140–230 CE (Flohr et al., 2017). However, it is also exemplified at Katalekhor, Kuna Ba, Jiroft, Hoti, and, as a result, in the centurial average z-score (Fig. 4). A short-lived amelioration of climate is then evidenced in the Kuna Ba and Gejkar records, before a return to arid conditions preceding the Sasanian takeover from the Parthians (200–220 CE). For the remainder of the third and the fourth century, effective moisture does not fluctuate rapidly and is relatively stable, with a slight upward trend at Gejkar and Kuna Ba and the inverse at Neor and Hoti (Fig. 4b). The centurial averages suggest an overall slightly wet third and fourth centuries, despite drier conditions at Neor and Gejkar (Fig. 4a). A rapid shift to much drier conditions from ~ 480 CE is arguably the most significant change observed in these records during the Sasanian Period. The Van, Neor, Katalekhor, and Hoti records suggest a period of significantly reduced effective moisture from ~ 480 to 540 CE (see full datasets in Supplementary Fig. S1). In the Neor and Hoti decadal averages, the 510s and 520s have the driest conditions, respectively. However, the Zeribar and Kuna Ba records suggest less pronounced drying that occurred ~ 540 CE. The centurial averages show a generally dry fifth and sixth century, despite this shift only coming at the end of the fifth century and not appearing in all records. There is further disagreement between the records in the sixth century, with the century average suggesting slightly dry conditions, but Katalekhor and Jiroft suggest much wetter conditions than in the previous century. The sixth century thus has the largest range of z-scores, both averaged across the whole period and in the 520s due to the delayed drying at Kuna Ba.

It is striking that two independently-dated high-resolution paleo-hydrological records, as well as several of the low-resolution records, suggest a dry phase that precedes the start of the “Late Antique Little Ice Age“ (LALIA) at 536 CE, a cool period in the northern hemisphere caused by aerosol ejected in three volcanic eruptions (Sigl et al., 2015; Büntgen et al., 2016). Drier conditions late in the fifth century CE are also indicated by pollen evidence from Lakes Gomishan, Maharlou and Parishan (Djamali, de Beaulieu, Miller et al., 2009; Leroy et al., 2013; Djamali et al., 2016; Saeidi Ghavi Andam et al., 2020; Matloubkari & Islam, 2022). While it is possible that the drying observed prior to 536 CE is due to dating inaccuracies and the reduction in effective moisture in all records is linked to the LALIA, this is highly unlikely, as it would require a similar and systematic error across numerous records with independent chronologies dated by different techniques (radiocarbon and uranium-series dating). Additionally, historical evidence supports earlier drying, with a peak in the number of references to droughts in SW Asia from 500–540 CE (McCormick et al., 2012; Fleitmann et al., 2022). Specifically, in Sasanian territories, there were events both before (e.g., famine in 527–529 CE) and during the LALIA (e.g., drought in 536 CE that ruined pasturage and forced a reported 15,000 Arabs to cross into Byzantine territory (Telelis, 2008; McCormick et al., 2012). Furthermore, high-resolution paleo-hydrological records in surrounding regions indicate a reduction in effective moisture decades prior to 536 CE. This extends to the Aegean (Skala Marion, Trichonida, Mavri Trypa (Finné et al., 2017; Psomiadis et al., 2018; Seguin et al., 2020)), Anatolia (Sofular, Kocain (Fleitmann et al., 2009; Jacobson et al., 2021)), the Levant (Jeita, Kfar Giladi (Cheng et al., 2015; Morin et al., 2019)), and Central Asia (Uluu-Too (Wolff et al., 2017)). Summer drying before 536 CE is also observed in central-east Europe and blamed for Hunnic raiding (Büntgen et al., 2021; Hakenbeck & Büntgen, 2022). However, it is worth noting that the only high-resolution record in Lower Mesopotamia covering this period (Kuna Ba) does not indicate pre-LALIA drying (Sinha et al., 2019).

After a brief wet phase around ~ 560 CE, another drier period is observed in the high-resolution records that ends ~ 620 CE at Neor and Hoti, and 670 CE at Kuna Ba. The seventh century overall experienced amelioration of conditions, being the wettest in terms of centurial average and during the 680s in the decadal average (Fig. 4). After the end of the Sasanian Empire, another period of low effective moisture is observed in many records between ~ 860 and 920 CE; at Kuna Ba, this extends to ~ 960 CE. This arid phase is especially pronounced and causes the ninth-century average z-score and 890–920 CE decadal averages to be the driest of the entire first millennium CE (Fig. 4). Sea sediment core OS73 from the Gulf of Oman also evidences the onset of drier conditions at this time, which may relate to the start of the Medieval Climate Anomaly (MCA) (Miller et al., 2016).

**Fig. 5:**
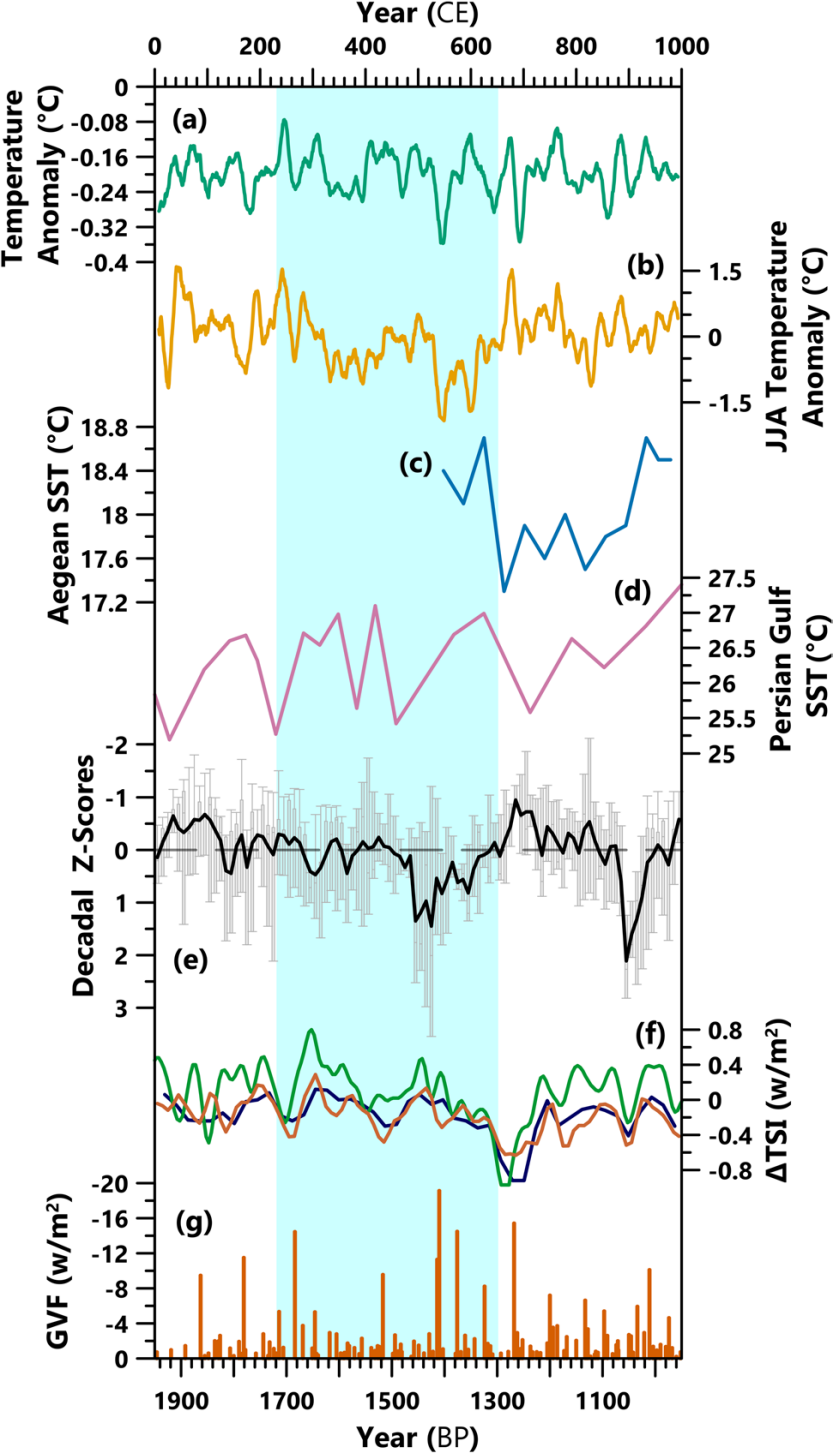
Comparison of decadal z-scores with records of temperature and climate-forcing mechanisms. (a) The 15-year average of global temperature anomaly record (PAGES 2k Consortium et al., 2019). (b) 15-year average of European Alps summer (June-August) temperature anomaly record (Büntgen et al., 2016). (c) Aegean SST reconstruction from sediment core M2 (Gogou et al., 2016). (d) Persian Gulf SST reconstruction from sediment core T2S3 (Safarkhani et al., 2021). (e) Box plot of decadal paleo-hydrological z-scores (from Fig. 4b), with mean values (black line). (f) Reconstructions of Total Solar Irradiance (TSI), displayed as deviations from the 1986 value of 1365.57 w/m-2: green (Steinhilber et al., 2009), brown (Vieira et al., 2011) and blue (Steinhilber et al., 2012). (g) Global Volcanic Forcing (GVF) from known volcanic eruptions (Sigl et al., 2015). It is important to consider that these datasets have varied age uncertainties that are not visualized here.

We compared the decadal z-scores with records of temperature and climate-forcing variables (volcanic eruptions and changes in solar irradiation) (Fig. 5). Overall, due to a lack of proximate records and the complexity of climate dynamics, causes for paleo-hydrological change in SW Asia are poorly understood. Tree-ring studies are principally used for high-resolution temperature reconstructions. However, due in part to the region’s aridity, these do not extend into the first millennium CE in SW Asia (Luterbacher et al., 2012). Within the region, there is one sea surface temperature (SST) reconstruction from marine core T2S3 in the Persian Gulf (Safarkhani et al., 2021) and another nearby from core M2 in the Aegean Sea (Gogou et al., 2016) (Fig. 5c, d), both low-resolution. Two other high-resolution temperature reconstructions are included for comparison; however, none of the 257 archives utilized by the PAGES 2k Consortium “Global” temperature anomaly dataset are from SW Asia (PAGES 2k Consortium et al., 2019), and the European Alps summer temperature dataset (Büntgen et al., 2016) is > 1,500 km outside the region. There is broad disagreement between the low-resolution temperature reconstructions from SW Asia and the high-resolution (but external) reconstructions. It is unclear whether the cause of these differences is actual climatic variability, chronological uncertainties in the marine sediment cores, the seasonal bias of tree-ring-based temperature reconstructions, or differences in the response times of land and sea temperatures. Three records of total solar irradiance (TSI) calculated from Greenland ice cores (Steinhilber et al., 2009; Vieira et al., 2011; Steinhilber et al., 2012) and a record of global volcanic forcing (GVF) from known eruption events (Sigl et al., 2015) are included. We observe no simple linear relationship between these records and the decadal z-scores. As stated, the dry conditions from ~ 480 CE pre-date the LALIA cluster of volcanic eruptions. They may correspond to warmer conditions, as evidenced in the high-resolution temperature reconstructions and one of the TSI records, but there is no evidence for these increased temperatures nearby. Furthermore, the wetter phase after the Sasanian Period (680–720 CE) is roughly synchronous to a period of low TSI, but so is the arid phase dated ~ 860–920 CE. Forcing mechanisms and temperature changes impact paleo-hydrological fluctuations variably, both temporally and spatially, contributing to the region’s high climatic spatial heterogeneity (see Jacobson et al., 2021).

## Climate and growth?

The Sasanian Period is the peak of all settlement surveys used in this study (Fig. 3) and the Khuzistan Plain surveys utilized by the Seshat databank (Adams, 1962; Wenke, 1987; Turchin et al., 2015). Archaeological and historical evidence suggest this was also the peak of irrigation construction, with an unprecedented expansion of river-fed canal systems, dams, weirs, and qanats (Gyselen, 2002; Campopiano, 2017; Manuel et al., 2018; Altaweel et al., 2019). The available pollen evidence suggests that the Sasanian Period was a peak of tree cultivation and cereal production in the region (Fig. 3), interpreted as resulting from political investment (Shumilovskikh et al., 2017). The empire also contributed to the development of colonies, such as in the Mughan Steppe, where military defenses with associated canal systems and settlements were constructed along the Araxes River (Alizadeh & Ur, 2007; Alizadeh, 2011; Ur & Alizadeh, 2013; Alizadeh et al., 2021).

The chronologies of these datasets are challenging (see below). However, current evidence indicates that Sasanian expansion and economic growth occurred during two main periods: first, the reign of Shapur II (309–379 CE), and second, the period from the restoration of Kavad I to the reign of Khosrau II (498–622 CE). Khosrau II’s reign ended in 628 CE, but from 622 CE, military losses and economic policy changes began to weaken the empire (Daryaee, 2022) (see below). This pattern is also reflected in the Seshat databank, with the largest increase in PC1 (“societal complexity”) observed in the fourth century CE, with two further large increases in the sixth and seventh centuries CE (Fig. 3). Before we can investigate the role of climate in these developments, we first explain what changes are defined as “growth” in each period:

The first growth phase aligns with the rule of Shapur II (309–379 CE), the longest-reigning monarch in Iranian history, who led successful campaigns in Arabia (Bosworth, 1999; Shayegan, 2004), against the Romans in Mesopotamia and the Mughan Steppe (Sicker, 2000; Daryaee & Rezakhani, 2016), and eastwards to Central Asia and India (Rezakhani, 2017). People from conquered territories were forcibly relocated to work in mines and on construction projects, many of which were completed in the fourth century CE (Morony, 2004; Shayegan, 2004). Large-scale military defenses (e.g., War-i Tāzigān, and Qal’eh Kharabeh) and cities (e.g., Bishapur, Nisibis, Susa) were constructed and rebuilt in new territories and core regions (Daryaee, 2009; Sauer et al., 2013, 2017). Cereal pollen percentages peaked at this time at Van, Almalou, and Bouara, indicating the intensification of agriculture (Van Zeist & Woldring, 1978; Gremmen & Bottema, 1991; Djamali, de Beaulieu, Andrieu-Ponel et al., 2009). Increased centralization and stratification of the Sasanian state and the completion of the Avesta, the sacred texts of Zoroastrianism, further contributed to the rise in PC1 observed in the Seshat dataset (Turchin et al., 2015; Daryaee & Rezakhani, 2017).

The second growth phase (498–622 CE) is linked to the restructuring of the Sasanian Empire by Kavad I (498–531 CE) and Khosrau I (531–579 CE) and expansion by Khosrau II (591–628 CE). The former designated Zoroastrian fire temples as economic and administrative institutions (Payne, 2014; Daryaee & Rezakhani, 2017) that organized the construction and maintenance of cities, irrigation infrastructure, and military defenses (Manuel et al., 2018; Maresca, 2019), e.g., forts at Fulayj and Ultan Qalası (Alizadeh, 2011; Al-Jahwari et al., 2018). Taxes on irrigation usage funded military campaigns and construction projects, (Gyselen, 1998; Khaneiki & Al-ghafri, 2022), a fixed agricultural tax that gave the Sasanian treasury a predictable income (Axworthy, 2008; Schindel, 2013a; Canepa & Daryaee, 2018) and expansion of Sasanian trade networks, including blocking Byzantine trade (Seland, 2012; Fakhar & Hesari, 2013; Howard-Johnston, 2017; Payne, 2018). This is exemplified by the proliferation of fifth-seventh century CE Sasanian silver coins across the Eastern Mediterranean and SW Asia (Schindel, 2013b), and further afield, e.g., Central Asia (Li, 2021), China (Li, 2006) and Britain (Abdy & Williams, 2006), as well as the dissemination of Sasanian commodities as far as Japan (Priestman, 2016). The increased wealth, as well as social and military reforms, enabled successful military campaigns and expansion (Sicker, 2000; Cameron, 2012). Continued conflict under Khosrau II led the Sasanian Empire to its greatest spatial extent between 619 and 628 CE (Fig. 1), including Yemen, Mesopotamia, the Levant, and Egypt (Yule, 2007; Cameron, 2012; Bowersock, 2013; Daryaee & Rezakhani, 2017).

Disentangling the factors contributing to these two growth phases is challenging. However, the role of climate appears negligible. The first growth phase occurred during a relatively stable period of effective moisture, with a slightly wet fourth-century z-score (Fig. 4), and persistently colder conditions suggested by the global and Alpine temperature reconstructions (Büntgen et al., 2016; PAGES 2k Consortium et al.,^ 2019^). It could be tempting to argue that the stable hydro-climate and colder conditions enabled the first growth phase through increased agricultural productivity, as reflected in the cereal maxima in many pollen records. However, the second growth phase occurs alongside the driest (and possibly coldest) conditions experienced by the Sasanian Empire. The same dry conditions around 490–550 CE contributed to a significant decline in the Kingdom of Himyar, with its capital in Zafar (Fleitmann et al., 2022; Haldon & Fleitmann, 2023). This brings into question the different situation in the Sasanian Empire that enabled it to continue unabated – or even thrive – under the same conditions, at least in some regions. It is possible that some parts of the Sasanian Empire were less susceptible to the drought, for example, in the north where rainfall is higher (Fig. 2), or were not impacted, for example, in the Fertile Crescent where the Kuna Ba record does not indicate pre-LALIA drying (Sinha et al., 2019). However, this would be surprising given the strong evidence for this in the Aegean, Anatolia, Levant, and Central Asia, all areas also under the influence of westerlies, and does not account for the Sasanian territories that were undeniably impacted. This implies that some characteristics of the Sasanian Empire made it resilient to climate change. Two important properties of a resilient system are (1) omnivory, a diversity of resources and resource-obtainment strategies, and (2) flexibility, meaning a system can rapidly adjust if required (Wildavsky, 1988; Barnett, 2001; Jacobson, 2022a, b). The available pollen and archaeological evidence, as well as the Babylonian Talmud, indicate varied agricultural strategies were adopted across Sasanian territories, with a wide range of crops, animal products, and shifting land-use patterns (Elman, 2004; Mashkour et al., 2017; Shumilovskikh et al., 2017; Adamo & Al-ansari, 2020; Panahipour, 2021). Perhaps more important for their resilience was the organized installation, repair, use, and maintenance of irrigation structures (Gyselen, 2002; Payne, 2014; Manuel et al., 2018).

These investments ensured that large areas of previously non-arable land were now cultivable and increased agricultural productivity across the Sasanian Empire. These developments, which may have been responses to already dry conditions, possibly buttressed against increasing aridity. An important distinction between the Sasanian Empire and the Kingdom of Himyar, which may have altered their relative resilience, is the water infrastructure primarily utilized. Qanats, which utilize gravity transport of water from upland regions to arid plains through underground tunnels, were abundant in the Sasanian Empire, especially in the Central Plateau (Beaumont, 1971; Semsar Yazdi & Semsar Yazdi, 2020). They are less sensitive to hydroclimatic fluctuations, as they draw water from aquifers rather than rainfall, and, due to their subterranean nature, are resistant to evaporation. This allows a near-continuous flow that only varies slightly between wet and dry years, thus providing water buffer (a key resilient characteristic) for use during drier years (Wildavsky, 1988; Lightfoot, 1996; Manuel et al., 2018). While a system similar to qanats is utilized in the modern Arabian Peninsula (there named *ghuyūl*), water infrastructure in the Kingdom of Himyar was almost entirely above ground, resulting in significantly more water loss through evaporation (Manuel et al., 2018; Haldon & Fleitmann, 2023). Furthermore, while qanats require careful management, a relatively small workforce can perform this compared to the considerable numbers required to maintain and repair terraces and dams (Harrower & Nathan, 2018).

In summary, as agriculture was the primary component of the Sasanian economy and the Empire contained exceptionally hot and dry regions, hydro-climatic fluctuations had the potential to cause significant damage. However, careful management of irrigation infrastructure, diverse agro-pastoral strategies and techniques, export of non-agricultural products, and import/movement of foodstuffs may have mitigated some negative impacts.

### Climate and Decline?

**Fig. 6:**
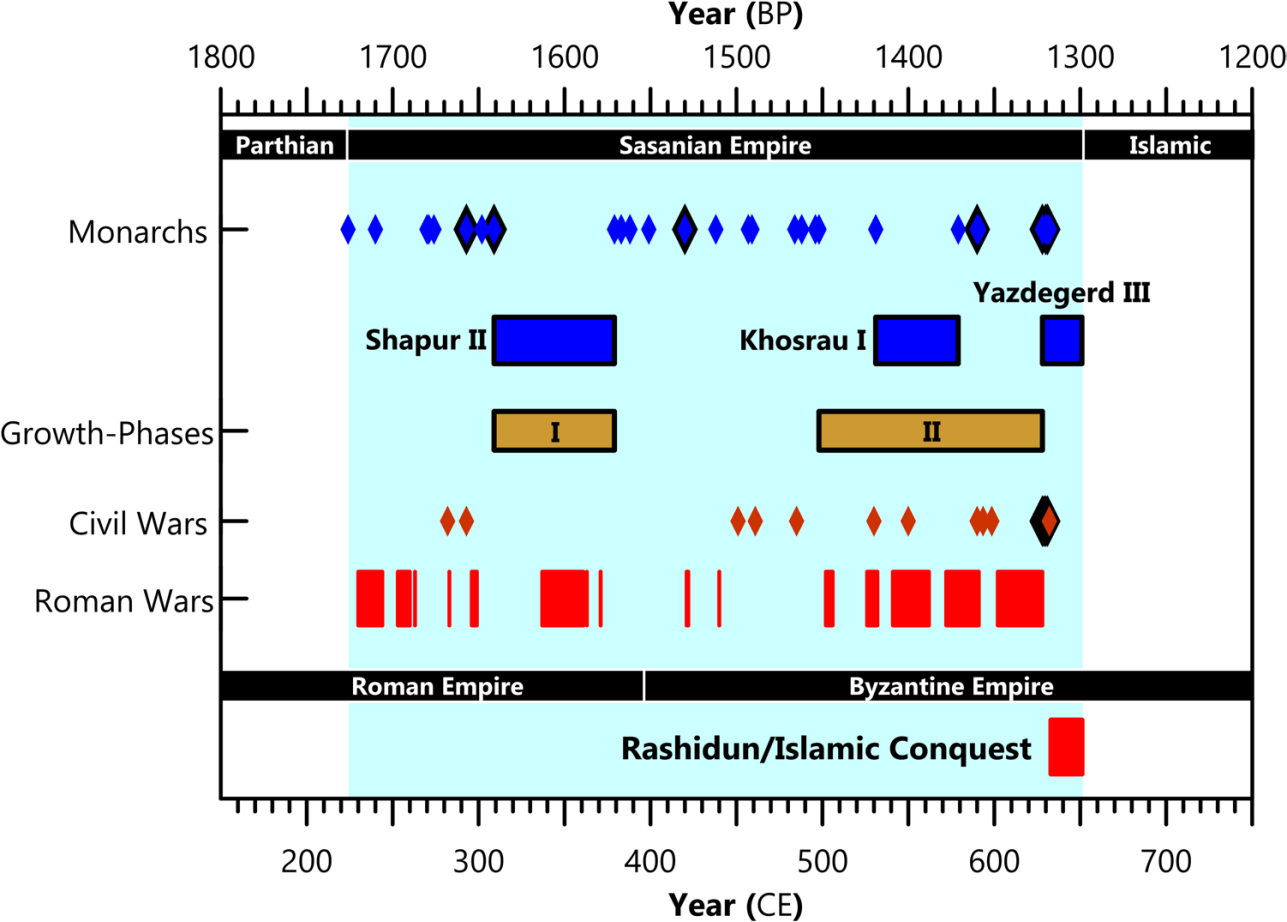
Timeline of the Sasanian Empire (dates from Daryaee & Rezakhani, 2016). Years with multiple monarchs/civil wars are highlighted by a black diamond

From 622 CE, the Sasanian Empire experienced rapid economic decline, with an associated loss of stability and manpower, associated with a counter-offensive launched from Constantinople by the Byzantine Heraclius (Daryaee, 2022). In response to Byzantine incursions, Khosrau II’s son, Shērōē/Kavad II (628 CE) seized power and executed his father and brothers (Payne, 2014; Daryaee & Rezakhani, 2016). A few months later, he died in an outbreak of the plague (Shērōē’s Plague: 627/8 CE), and during the civil war that followed, 11 monarchs took the throne between 628 and 632 CE (Daryaee & Rezakhani, 2016; Shahraki et al., 2016) (see Fig. 6). This conflict culminated with Yazdegerd III (632–651 CE) inheriting the throne at eight years old; however, many regional kings and governors had proclaimed independence, and the empire had returned to a political system akin to the decentralized Parthians (Pourshariati, 2008; Kia, 2016). This fragmentation emboldened attacks from the Göktürks in Central Asia and Khazars in the Caucasus, contemporaneous to invasions by the Rashidun Caliphate (Wiesehöfer, 2010). Yazdegerd III was eventually murdered in 651 CE in the city of Merv while attempting to flee the invading Rashidun Caliphate (Wiesehöfer, 2010), signaling the end of the Sasanian Empire.

The actual impact of these developments on communities within Sasanian territories has been contested (e.g., Soroush, 2020). For example, the Pahlavi letters suggest that ordinary people near modern Tehran continue living as before in the seventh-ninth centuries (Weber, 2012). However, there is a reduction in settlement numbers in all archaeological surveys (Fig. 3), and tax records indicate a significant reduction (~ 50%) in revenue in Lower Mesopotamia from the sixth to seventh centuries (Christensen, 1993; Daryaee, 2022). In the Persian Gulf, the reduction in site numbers at Bushehr (Carter et al., 2006) is mirrored in other nearby districts (Askari Chaveri & Azarnoush, 2004; Asadi, 2010, 2013). However, it is less extreme in other regions, such as the Kur River basin (Hartnell, 2014) (Fig. 3). Following the Muslim conquests, the abandonment of fortified sites in this region, which acted as administrative centers for local agriculture, is suggested to have caused the reduction of settlement numbers (Asadi et al., 2013). Abandonment of fortifications was widespread, for example, at Ultan Qalası in the Mughan Steppe (Alizadeh et al., 2021), as was a decline in irrigation infrastructure. Deterioration of infrastructure was previously interpreted as a cause of decline; however, it is now better understood that a failure to recover from damage is a symptom of strife as a community’s ability to organize and mobilize the required large workforce is undermined (Asadi et al., 2013; Jacobson, 2022a, b). Another Late Antique example of this occurred in the Arabian Peninsula, with the abandonment of the dam of Ma’rib following the dissolution of the Kingdom of Himyar (Harrower & Nathan, 2018; Fleitmann et al., 2022). In contrast, Mehrnoush Soroush convincingly argues that infrastructure was less influenced by the invasions than is often presumed, with investment continuing in Lower Mesopotamia throughout the Sasanian Period and beyond (Soroush, 2014, 2020). She suggests that a gradual decline in the quality of irrigation infrastructure occurred much later, in the ninth-tenth centuries CE, associated with down-cutting of riverbeds that increased maintenance costs (Soroush, 2020).

As discussed above, the driest climatic conditions during the Sasanian Period pre-date the Empire’s decline (from 628 CE) and end (651 CE) by over a century. During the decline, many paleoclimate proxy records indicate that conditions were getting wetter, with the seventh century CE having the wettest centurial z-score (Fig. 4). However, Kuna Ba, the only high-resolution archive covering this period in Lower Mesopotamia, does indicate slightly drier conditions 620–680 CE (Sinha et al., 2019). From about 640 CE, the closer SST records suggest cooling (Gogou et al., 2016; Safarkhani et al., 2021), coincident with a reduction in total solar irradiance (Steinhilber et al., 2009; Vieira et al., 2011; Steinhilber et al., 2012) (Fig. 5). Overall, current evidence suggests that climate was not a factor in the decline of the Sasanian Empire. As Daryaee (2022) recently explained, fighting wars on numerous fronts and internally, decline in agriculture and irrigation infrastructure, plague, and fragmentation of power to generals left the Sasanian state weakened and easily overrun when the Muslim armies attacked. For the Sasanian Empire, a coalescence of factors contributed to their decline, as has been observed in other regions in Late Antiquity (Korotayev et al., 1999; Fleitmann et al., 2022; Jacobson et al., 2022); however, in this instance, climate change does not appear to have been one of the decisive factors.

## Challenges

We have incorporated currently available archaeological, pollen, and paleoclimatic proxy evidence into the complex and detailed history of the Sasanian Empire. We now further examine the challenges in linking climate change to the Sasanian Empire’s development and suggest future research directions. One of the key challenges in studying Iranian archaeology is the division of research between English, other European, and Persian-language literature; this is also a key weakness of this study. As such, Iranian researchers may have already rectified some of the challenges we outline below in research that we are unable to access.

Despite nine paleo-hydrological records of varying quality and other indicators of past climatic changes (pollen, historical records) in the region, there are still notable gaps in our knowledge of climatic and environmental conditions during the Sasanian Period. The most prominent is that only one low-resolution paleo-temperature record is present due to environmental conditions. Therefore, we do not know how temperature evolved in shorter timescales and other areas. This is especially limiting, as the Persian Gulf sediment core T2S3 record does not match with the high-resolution global and Alpine temperature reconstructions (Büntgen et al., 2016; PAGES 2k Consortium et al., 2019; Safarkhani et al., 2021). Furthermore, there is no simple relationship between temperature and paleo-hydrological conditions, as has been noted elsewhere (e.g., Jacobson et al., 2021), with dry and wet phases accompanied by both hot and cold conditions under different circumstances (Fig. 5). Additionally, there are spatial and temporal gaps in the coverage of paleo-hydrological records. Most records are limited to the west due to their formation conditions, as they require adequate effective moisture to form, which is not present in the drier east. Two records, Jiroft (Vaezi et al., 2022) and Hoti (Fleitmann et al., 2022), are east of 50°E – the former is low-resolution and the latter is more appropriate for conditions over the Arabian Peninsula than Persia. Jiroft deviates from many of the other records (Fig. 4 and Supplementary Fig. S1), so finding a high-resolution archive nearby and to the north where there is a complete absence of records between 30° – 40° N and 50° – 70° E, is high-priority. In Lower Mesopotamia, the Kuna Ba record (Sinha et al., 2019) suggests the region experienced different climatic changes (Fig. 4), but more archives are required to determine the spatial extent of these differences.

Issues of chronology further complicate assessing the role of climate changes in the history of the Sasanian Empire. Paleoclimate records each have unique resolutions and chronological uncertainties, which can make displaying line graphs of the data misleading as these do not visualize such uncertainties. This is a significant challenge, but we can be more confident of paleoclimate changes when supported by multiple independently-dated records. Above, we discuss how the drying prior to 536 CE is unlikely to result from uncertainties given the large number of supporting records, both internal and external to the Sasanian Empire. The archaeological evidence presents bigger chronological challenges, partially resulting from a bias toward researching the Achaemenid and Islamic periods in Iranian history (Genito, 2016). Unfortunately, settlement surveys, which date identified settlements and associated irrigation infrastructure primarily with ceramics, rarely provide chronologies that are more precise than just ‘Sasanian’ (Fig. 3). Other ceramics are designated to even broader chronologies that encompass the Parthian and/or the early Islamic periods (Mousavi & Daryaee, 2012; Neely, 2016). This is a common problem in archaeology and implies settlement occupation for an entire period, which may be accurate, but occupation could equally have been of short duration or discontinuous, restarting multiple times throughout the period (Plog, 1973; Dewar, 1991). Thus, sites that were not contemporaneous may appear as such (Schacht, 1984; Premo, 2014). Settlements could be better dated with tighter chronological ceramic typologies (e.g., Kennet, 2002), toponymal analysis (many cities were named after the kings that (re-)built them, e.g., Bishapur (Karimian, 2010), or assessment of coin finds (Mousavi & Daryaee, 2012). However, much of such work has not yet been completed. Additionally, a lack of widespread use of scientific dating methods in the archaeology of the Sasanian Empire hinders our ability to understand past climate-society interactions. This issue is common across the Antique and Medieval periods, for example, in studies of the Roman Eastern Mediterranean. Troubles with precise chronology make assessing factors behind the growth and decline especially challenging. For example, the abandonment of the Mughan Steppe and the decline of irrigation in Lower Mesopotamia could have occurred before, during, or after the Islamic conquests (Soroush, 2014; Alizadeh et al., 2021). Though there are a few pollen records with numerous data points during the Sasanian Period (Fig. 3), most only contain one or two samples, which limits understanding. Furthermore, excluding Lake Van, which is dated by varve counts (Van Zeist & Woldring, 1978); the pollen records are dated by varying numbers of radiocarbon dates, which have large chronological uncertainties (often ± centuries). While the Sasanian Period appears to be the peak of arboriculture and cereal cultivation in most pollen records (Shumilovskikh et al., 2017), it is hard to say this for sure and particularly to identify more precisely when this was.

An additional challenge is understanding the influence of specific climate changes, how their impacts are lessened or intensified by the community/society being studied, and baseline environmental conditions. Assumptions are often made in studies of ancient climate-society interactions that do not appear valid for the Sasanian Empire. One example is the theory that cool temperatures have more severe negative impacts than warming (McMichael, 2012; Büntgen et al., 2016), with warmer and wetter conditions being advantageous to agricultural productivity (Harper, 2017). However, the second Sasanian growth period occurred during the driest (possibly also colder) conditions and the subsequent decline coincided with increasing moisture. It seems likely that climate was not a driver for these developments. However, it is also likely that the supposed impacts of specific changes would be different in the varied regions of the Sasanian Empire, especially given the high temperatures and aridity of some regions. In such environments, cooler temperatures may be more advantageous as increased effective moisture, resulting from reduced evaporation, promotes plant growth (Gohari et al., 2013). Furthermore, perhaps due to the persistent aridity, the Sasanians (and their predecessors) were already reliant on hydraulic infrastructure and diverse agro-pastoral strategies to overcome a deficiency of effective moisture (Seyf, 2006; Shumilovskikh et al., 2017; Soroush, 2020), which may have reduced the impacts of a multi-year drought. Similarly, northern regions that experience high precipitation (e.g., 1181 mm/yr at Lenkoran: see Fig. 2), may have been relatively insensitive to hydro-climatic fluctuations.

In summary, there are still many gaps in our knowledge regarding the Sasanian Empire and past climate changes; these currently hinder our ability to make confident conclusions about their interactions. A productive next step would be to study Sasanian climate-society interactions in a much smaller case-study region, selected based on the availability and quality of datasets. This approach has proved productive elsewhere, such as in Greece (Weiberg et al., 2021) and Turkey (Jacobson et al., 2022). Currently, Lower Mesopotamia (or Khuzistan for a smaller case study) is perhaps best suited to this type of assessment, although it would benefit from an improved archaeological chronology and a closer pollen record.

## Conclusion

We collated a wide range of archaeological, historical, paleoclimatic, and pollen data to assess whether climate change played a role in the growth and decline of the Sasanian Empire. Based on the currently available data, we suggest that climate change did not play a significant role in these developments, with the Empire expanding during two periods with very different conditions. The first (309–379 CE) period had stable effective moisture and cooler temperatures, tempting a climatic interpretation. However, the second (498–622 CE) was the driest phase in the Sasanian Empire’s history – the same dry phase that caused detrimental effects in other regions. We suggest that the prevalence of qanats, careful management of irrigation infrastructure, as well as diverse agro-pastoral strategies and techniques (which were possibly necessitated by the region’s predominantly hot/dry conditions) made the Sasanian Empire more resilient in the face of this long-lasting drought.

However, in the last section, we emphasize three key gaps in our knowledge that currently preclude confident conclusions. First, detailed evidence of how and on what spatial scales climatic conditions changed is still lacking. No high-quality paleo-temperature records are relevant for the Sasanian Empire, making understanding the influence of temperature on societal changes impossible. The Kuna Ba record from Lower Mesopotamia also shows different climatic changes to those in surrounding regions, so more research is required to ascertain the spatial extent of these differences. Second, the chronologies and resolutions of archaeological and pollen datasets need to be improved. Especially in settlement archaeology, data only suggest a peak during the Sasanian Period (and in some surveys, the Parthian-Sasanian or Sasanian-early Islamic periods). This tells us nothing about when the growth or decline of settlements occurred and makes assessing the relevance of different factors challenging. Finally, there is uncertainty about what influence should be expected from specific climate changes. The impact of climatic shifts on agriculture and society will differ depending on the baseline environmental conditions of the study region and the practices of the communities living there. Overall, further research is required to confirm our conclusions or those of Matloubkari and Islam (2022).

## Supplementary Information

Below is the link to the electronic supplementary material.

ESM 1 (225 MB)

## Data Availability

No datasets were generated or analysed during the current study.
